# Immunophenotyping with (phospho)protein profiling and fluorescent cell barcoding for single-cell signaling analysis and biomarker discovery

**DOI:** 10.1038/s41698-024-00604-y

**Published:** 2024-05-20

**Authors:** Johanne U. Hermansen, Yanping Yin, Idun Dale Rein, Sigrid S. Skånland

**Affiliations:** 1 Department of Cancer Immunology, Institute for Cancer Research, Oslo University Hospital, Oslo, Norway; 2 K. G. Jebsen Centre for B Cell Malignancies, Institute of Clinical Medicine, University of Oslo, Oslo, Norway; 3 Department of Radiation Biology, Institute for Cancer Research, Oslo University Hospital, Oslo, Norway

**Keywords:** Lymphocytes, Biomarkers

## Abstract

The microenvironment of hematologic cancers contributes to tumor cell survival and proliferation, as well as treatment resistance. Understanding tumor- and drug-induced changes to the immune cell composition and functionality is therefore critical for implementing optimal treatment strategies and for the development of novel cancer therapies. The liquid nature of peripheral blood makes this organ uniquely suited for single-cell studies by flow cytometry. (Phospho)protein profiles detected by flow cytometry analyses have been shown to correlate with ex vivo drug sensitivity and to predict treatment outcomes in hematologic cancers, demonstrating that this method is suitable for pre-clinical studies. Here, we present a flow cytometry protocol that combines multi-parameter immunophenotyping with single-cell (phospho)protein profiling. The protocol makes use of fluorescent cell barcoding, which means that multiple cell samples, either collected from different donors or exposed to different treatment conditions, can be combined and analyzed as one experiment. This reduces variability between samples, increases the throughput of the experiment, and lowers experimental costs. This protocol may serve as a guide for the use and further development of assays to study immunophenotype and cell signaling at single-cell resolution in normal and malignant cells. The read-outs may provide biological insight into cancer pathogenesis, identify novel drug targets, and ultimately serve as a biomarker to guide clinical decision-making.

## Introduction

A cancer cell depends on signals from its microenvironment to survive and proliferate in the host organism^1^. For hematologic cancers, the microenvironment is composed of peripheral blood, bone marrow, lymph nodes, and secondary lymphoid organs. It is known that the tumor microenvironment also contributes to treatment resistance^2–4^, underscoring the need to understand its composition and functionality so that effective treatment strategies can be developed.

The liquid nature of peripheral blood makes this organ uniquely suited for single-cell studies by flow cytometry, which allows for in-depth dissection of its composition as well as tumor-induced alterations. Technological advances including the development of high-parameter cell analyzers, a recent expansion of available fluorescent dyes, and continuous approval of targeted therapies that are compatible with ex vivo studies, have led to an explosive increase in the conceivable resolution of a single experiment as well as its clinical relevance. Single-cell flow cytometry studies have contributed to an improved understanding of tumor biology and ex vivo drug responses^5–9^, demonstrating that the method is suitable for pre-clinical studies.

Precision medicine is often associated with genomic profiling. However, it can also be guided by functional analyses of the patient’s tumor cells^10–14^. (Phospho)protein profiles detected by single-cell signaling analyses have been shown to correlate with ex vivo drug sensitivity and to predict treatment outcomes in hematologic cancers^15–17^. Neither genomic nor functional precision medicine can identify optimal treatment strategies for every patient. However, it is likely that integration of the two approaches will identify actionable drug targets for a larger number of cancer patients. By improving and expanding on available diagnostic tools, we will more likely succeed with ensuring the best possible treatment predictions and clinical decisions for each patient.

Here, we present a single-cell flow cytometry protocol that combines multi-parameter immunophenotyping with single-cell (phospho)protein profiling. The protocol makes use of fluorescent cell barcoding (FCB), which means that multiple cell samples, either collected from different patients or exposed to different treatment conditions, can be combined and analyzed as one experiment^18^. This reduces variability between samples^19^, increases the throughput of the experiment, and lowers experimental costs. The protocol may serve as a guide for the use and further development of assays to study immunophenotype and cell signaling at single-cell resolution in normal and malignant cells, and the results may identify biomarkers that can guide functional precision medicine.

## Results

### Protocol for immunophenotyping with (phospho)protein profiling

Below, we present a protocol that combines immunophenotyping and (phospho)protein profiling of PBMCs from healthy donors or patients with hematologic cancer. The protocol requires access to a high-parameter cell analyzer (see “Flow cytometry analysis” for details). We present the protocol as it has been optimized in our laboratory for PBMCs from healthy donors and chronic lymphocytic leukemia (CLL) patients as a starting point for experimental setups. Antibody titrations, staining procedures, and sample handling should be optimized by each user and adapted to the sample type. The antibodies used herein are validated by the manufacturers for the species and application.

**Fig. 1:**
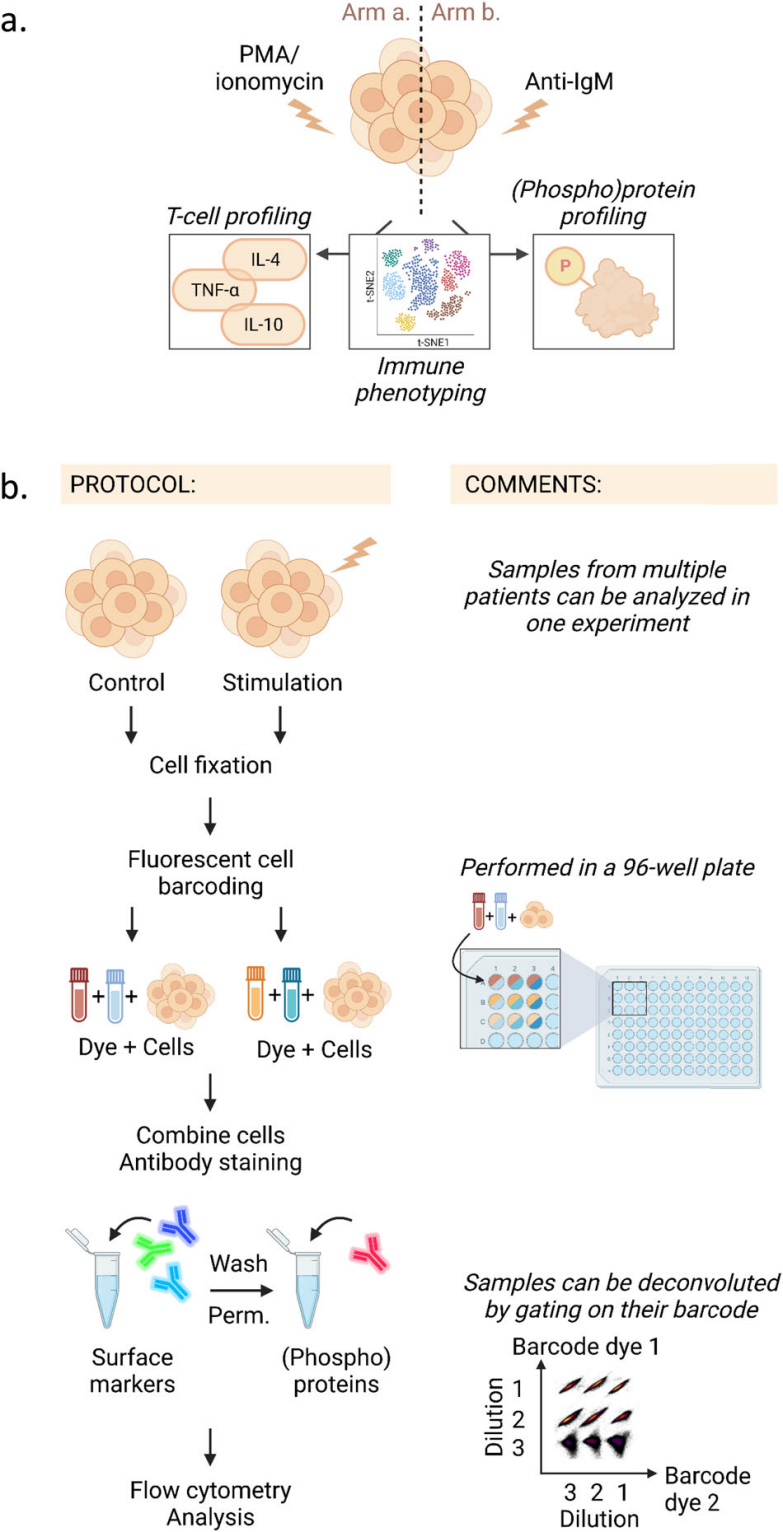
Schematic presentation of the protocol. **a** Simplified illustration of the protocol. The cell sample is divided in two (Arm a and Arm b). Each sample is immunophenotyped. The sample in Arm a is subjected to T-cell stimulation and T-cell profiling (left panel). The sample in Arm b is subjected to B-cell stimulation and (phospho)protein profiling (right panel). Both samples are analysed with a high-parameter flow cytometer. **b** Illustration of the step-by-step protocol. The cells are stimulated before cell fixation. Fluorescent cell barcoding is then performed so that all the samples can be combined in one tube before antibody staining. The cells are first stained with antibodies against surface markers, then washed and permeabilized before staining with antibodies against intracellular (phospho)proteins. The cells are analysed with a flow cytometer. Barcoded samples can be deconvoluted by gating on their barcodes. The figure was created in BioRender.

The PBMCs are analyzed in two experimental arms (Fig. 1a). The cells in Arm a are exposed to T-cell stimulation, then immunophenotyped, and profiled for cytokine expression and (phospho)protein levels (Fig. 1a, left). The cells in Arm b are exposed to B-cell stimulation, then immunophenotyped and profiled for (phospho)protein levels (Fig. 1a, right).

### Thawing and preparation of PBMCs

**NOTE:** The experiments can be performed on fresh or frozen PBMCs. Below, we describe the procedure when starting with frozen PBMCs. For this protocol, 22–27 × 10^6^ PBMCs are needed.

1. Quickly thaw the cells in a 37 °C water bath
2. Wash the cells once with 10 mL Roswell Park Memorial Institute (RPMI) 1640 medium supplemented with GlutaMAX and 1x sodium pyruvate, 1x MEM non-essential amino acids, 1x penicillin/streptomycin, and 10% fetal bovine serum (FBS) (complete RPMI)
3. Centrifuge the cells at 300 x *g* for 5 min at room temperature (RT). Discard the supernatant
4. Resuspend the cells in complete RPMI
5. Count the cells using the preferred method and distribute each sample as follows into separate 1.5 mL tubes:
  - 10 × 10^6^ cells for phorbol myristate acetate (PMA)/ionomycin stimulation (analyzed in “T-cell stimulation with PMA/ionomycin and cell fixation (Arm A)”)
  - 10 × 10^6^ cells for anti-IgM stimulation (analyzed in “B-cell receptor (BCR) stimulation with anti-IgM antibody and cell fixation (Arm b)”)
  - 5 × 10^6^ cells for FCB compensation controls (analyzed in “Fluorescent cell barcoding”)
    **NOTE:** It is possible to collect left-over cells from different donors for this control sample
  - 2 × 10^6^ cells for an unstained control (analyzed in “Flow cytometry analysis”)

### T-cell stimulation with PMA/ionomycin and cell fixation (Arm a)

**NOTE:** Sections “T-cell stimulation with PMA/ionomycin and cell fixation (Arm a)” and “B-cell receptor (BCR) stimulation with anti-IgM antibody and cell fixation (Arm b)” are performed in parallel.

**CAUTION:** Fix Buffer I contains formaldehyde which is toxic (skin contact) and a potential carcinogen. Handle with care.

1. Divide the cells collected for PMA/ionomycin stimulation (“Thawing and preparation of PBMCs”, a) into two 15 mL tubes:
  - 5 × 10^6^ cells for Brefeldin A (BFA) treatment (control)
  - 5 × 10^6^ cells for BFA + PMA/ionomycin treatment (stimulation)

1. Add treatment reagents to each tube according to Table 1 and adjust the total volume to 2500 µL with complete RPMI

**Table 1:** Reagents for B- and T-cell activation

Reagent	Vendor	Cat. no	Stock solution	Final concentration	Intermediate dilution (in complete RPMI)	Added to the cell suspension
Goat F(ab’)2 Anti-Human IgM-UNLB	Southern Biotech	2022-01	0.5 mg/mL	10 μg/mL	–	1:50
Brefeldin A	Sigma-Aldrich	B5936	10 mg/mL	10 μg/mL	1:10	25 μL
Complete RPMI	VWR	392–0428	–	–	–	Up to 2500 μL
Ionomycin	Sigma-Aldrich	I0634-1MG	1 mg/mL	1 μg/mL	1:10	25 μL
Phorbol 12-myristate 13-acetate (PMA)	Sigma-Aldrich	P1585	10 mg/mL	10 ng/mL	1:10 000	25 μL
2. Leave the tubes with the cap open in an incubator at 5% CO_2_, 37 °C for 4 h
3. Wash the cells twice with phosphate-buffered saline (PBS) supplemented with 2% FBS (flow wash)
4. Centrifuge the cells at 300 x *g* for 5 min at RT. Discard the supernatant
5. Stain each cell pellet with live/dead fixable blue (Cat. no. L23105; Thermo Fisher Scientific, Waltham, MA, USA; 1:8000 dilution in PBS). Leave for 15 min at RT in the dark
6. Wash the cells twice with flow wash. Centrifuge the cells at 300 x *g* for 5 min at RT. Discard the supernatant
7. Resuspend the cells in 100 µL flow wash
8. Fix the cells by adding 100 µL Fix buffer I (Cat. no. 557870; BD Biosciences) to the cell suspension. Mix by pipetting up and down 2–3 times
  Fix and wash the cells to be used as control samples (“Thawing and preparation of PBMCs”, c-d) in parallel. These will be processed further in “Fluorescent cell barcoding” and “Flow cytometry analysis”.
9. Leave for 10 min at RT in the dark
10. Wash the cells twice with PBS. Centrifuge at 500 x *g* for 5 min at RT. Discard the supernatant
11. Proceed to «Fluorescent cell barcoding”

### B-cell receptor (BCR) stimulation with anti-IgM antibody and cell fixation (Arm b)

1. Centrifuge the cells (“Thawing and preparation of PBMCs”, b) at 300 x *g* for 5 min at RT. Discard the supernatant
2. Stain the cells with live/dead fixable blue (Cat. no. L23105; Thermo Fisher Scientific, Waltham, MA, USA; 1:8000 dilution in PBS) in a 1.5 mL tube
3. Leave for 15 min at RT in the dark
4. Wash the cells twice with flow wash. Centrifuge the cells at 300 x *g* for 5 min at RT. Discard the supernatant
5. Resuspend the cells in 100 µL complete RPMI. Incubate in a 37 °C water bath for 10 min
6. Stimulate the cells with anti-IgM antibody and fix:
  - Prepare a 96-well V-bottom plate with 60 µL Fix Buffer I per well per sample. Leave it in the 37 °C water bath
  - Transfer 50 µL of the cell solution (control; 0 min) to the fix plate and mix well by pipetting up and down 2–3 times
  - Start the stimulation by adding anti-IgM (10 µg/mL) to the remaining cell solution and mix well by pipetting up and down 2–3 times. Leave the cells in the 37 °C water bath and incubate for 5 min
  - Transfer the remaining 50 µL of the cell solution (stimulated; 5 min) to the fix plate and mix well by pipetting up and down 2–3 times
  - Fix the cells by leaving the plate in the 37 °C water bath for 10 min
7. Wash the cells in the plate twice with PBS. Centrifuge at 500 x *g* for 5 min at RT. Discard the supernatant
8. Proceed to “Fluorescent cell barcoding”

### Preparation of internal control

**NOTE:** The internal control is used as a reference for the intracellular (phospho)protein signals in the test samples. It is recommended to use PBMCs from a donor with a high number of cells as the control sample so that the same control can be used in multiple experiments. When the donor material for the internal control is starting to run out, a new internal control donor needs to be run together with the old donor in a barcode matrix for each (phospho)protein. This will make it possible to establish a factor for normalizing the signals across signal variations in internal control donors.

1. Quickly thaw the PBMCs in a 37 °C water bath
2. Wash the cells once with 10 mL of complete RPMI
3. Centrifuge the cells at 300 x *g* for 5 min at RT. Discard the supernatant
4. Stain the cells with live/dead fixable blue (Cat. no. L23105; Thermo Fisher Scientific, Waltham, MA, USA; 1:8000 dilution in PBS)
5. Leave for 15 min at RT in the dark
6. Wash the cells twice with flow wash
7. Centrifuge the cells at 300 x *g* for 5 min at RT. Discard the supernatant
8. Resuspend the cells with 100 µL flow wash
9. Fix the cells by adding 100 µL Fix buffer I to the cell suspension. Mix well by pipetting up and down 2–3 times
10. Leave for 10 min in a 37 °C water bath
11. Wash the cells twice with PBS. Centrifuge the cells at 500 x *g* for 5 min at RT. Discard the supernatant
12. Resuspend the cells with flow wash
13. Aliquot the cells into 5 million cells per vial and store the cells at - 80 °C until use

### Fluorescent cell barcoding

**NOTE:** Samples from “T-cell stimulation with PMA/ionomycin and cell fixation (Arm a)” and “B-cell receptor (BCR) stimulation with anti-IgM antibody and cell fixation (Arm b)” are stained with barcoding reagents separately. Include one internal control (“Preparation of internal control”) in each barcoding matrix.

1. Wash the cells once with PBS containing 0.02% saponin, as previously reported^20^
  **NOTE**: Saponin is temperature and time sensitive and should be kept at 4 °C for maximum 1 week. Stock solution (10%) can be stored at −20 °C long term.
2. Centrifuge the cells at 500 x *g* for 5 min at 4 °C. Discard the supernatant
3. Resuspend each sample in 190 µL PBS with 0.02% saponin on ice
4. FCB:
  - Prepare barcoding reagents (see Table 2) in a 96-well V-bottom plate by pipetting 5 µL of each barcoding reagent per well in the number of combinations required to stain all samples following the staining matrix, e.g., Fig. 1b

**Table 2:** Barcoding reagents

Barcoding reagent	Vendor	Cat. no.	Stock solution (in DMSO)	Serial dilutions (starting with the stock solution)		
				#1	#2	#3
Pacific Blue	Thermo Fisher Scientific	P10163	10 mg/mL	1:2500	1:4	1:40
Pacific Orange	Thermo Fisher Scientific	P30253	2 mg/mL	1:50	1:12	1:240
  - Transfer each cell sample to the appropriate well on the barcoding plate (Fig. 1b). Mix thoroughly by pipetting up and down 2–3 times
    Stain one compensation sample (“Thawing and preparation of PBMCs”, c) with the highest final concentration used for each barcoding reagent (one sample/dye) and save one unstained sample.
  - Leave the cells for 20 min at RT in the dark

6. Wash the cells twice with flow wash
7. Centrifuge the cells at 500 x *g* for 5 min at RT. Discard the supernatant
8. Resuspend the cells in 150 µL flow wash and combine the barcoded samples in one 1.5 mL tube. Transfer each compensation control to a separate 1.5 mL tube and add an equivalent number of unstained cells to each compensation control tube
9. Centrifuge at 500 x *g* for 5 min at RT. Discard the supernatant

### Surface antigen staining and cell permeabilization

**NOTE:** The barcoded samples from Arm a and Arm b are handled separately.

**NOTE:** The staining protocol has been optimized for best signal detection for each antibody in Table 3. The PBMCs are stained with all antibodies, except anti-CD16 and anti-FoxP3, before permeabilization.

**Table 3:** Antibody panel for immunophenotyping

Antigen	Fluorochrome	Vendor	Cat. no.	Volume
CD3	BUV395	BD Biosciences	563546	1 µL
CD4	BUV563	BD Biosciences	612913	2 µL
CD8	BV786	BD Biosciences	563825	1 µL
CD16^a^	BUV737	BD Biosciences	612787	1 µL
CD19	APC-Cy7	BD Biosciences	557791	5 µL
CD25	PE-CF594	BD Biosciences	562525	5 µL
CD45RA	PE-Cy7	BD Biosciences	560675	1 µL
CD56	BV750	BD Biosciences	747068	1 µL
CD69	APC-R700	BD Biosciences	565154	1 µL
CD183	PE-Cy5	BD Biosciences	561731	5 µL
CD185	BUV805	BD Biosciences	741980	1 µL
CD196	BV711	BD Biosciences	563923	1 µL
CD197	PE	BD Biosciences	560765	5 µL
FoxP3^a^	AF488	BD Biosciences	560047	5 µl
HLA-DR	BUV615	BD Biosciences	751142	1 µL
PD-1	BB700	BD Biosciences	566460	1 µL

^a^Used after cell permeabilization.

**PREPARATION:** Transfer 1 mL of Perm Buffer III (Cat. no. 558052; BD Biosciences) to a 15 mL tube. Leave at −20 °C so it is ice-cold upon use.

**CAUTION:** The main ingredient of Perm Buffer III is methanol which is toxic (inhalation and skin contact) and flammable. Handle with care.

1. Prepare the antibody mix for surface marker staining per sample according to Table 3 in a 1.5 mL tube. Add 10 µl Brilliant Stain Buffer (Cat. no. 566385; BD Biosciences) and adjust the total volume to 50 µL with flow wash. Add the surface marker mix to the cell pellet and mix well by pipetting up and down 2–3 times
2. Leave for 30 min at RT in the dark
3. Wash the cells twice with flow wash
4. Centrifuge the cells at 500 x g for 5 min at RT. Discard the supernatant
5. Permeabilize the cells in 200 µL Human FoxP3 Buffer (Cat. no. 560098; BD Biosciences)
6. Leave for 30 min at RT in the dark
7. Wash the cells once with flow wash
8. Resuspend the cells and the compensation controls (“Fluorescent cell barcoding”, 9) in 100 µL ice-cold Perm Buffer III on ice. Cells in Perm Buffer can be stored long term at - 80 °C

**NOTE:** It is natural to pause the experiment here.

### Intracellular antigen staining of PMA/ionomycin stimulated samples (Arm a)

**NOTE:** Perform Sections “Intracellular antigen staining of PMA/ionomycin stimulated samples (Arm a)”, “Intracellular antigen staining of anti-IgM stimulated samples (Arm b)”, and “Preparation of compensation controls” in parallel.

1. Transfer the samples from - 80 °C to a box of ice
2. Wash the cells three times with flow wash
  **NOTE**: It is important to add flow wash in excess to see the cell pellet, e.g., add 1.5 mL of flow wash to the barcoded cell population and each compensation control.
3. Centrifuge the cells at 500 x *g* for 5 min at RT. Discard the supernatant
  **NOTE:** Collect the Perm Buffer III which contains methanol for appropriate waste disposal.
4. Prepare the antibody mix for anti-CD16 staining according to Table 3 in a 1.5 mL tube. Add 10 µl Brilliant Stain Buffer and adjust the total volume to 50 µL with flow wash. Add the surface marker mix to the cell pellet and mix by pipetting up and down 2–3 times
5. Leave for 30 min at RT in the dark
6. Wash the cells twice with flow wash
7. Resuspend the cells in 45 μl flow wash. Add 5 μl anti-FoxP3 to the suspension and mix by pipetting up and down 2-3 times
8. Leave for 30 min at RT in the dark
9. Wash the cells twice with flow wash
10. Centrifuge the cells at 500 x *g* for 5 min at RT. Discard the supernatant
11. Resuspend the cells in 40 µL flow wash per staining condition (i.e., 400 µL if using the panel of 10 antibodies listed in Table 4)

**Table 4:** Antibody panel for T-cell profiling

Antibody (AF647-conjugated)	Vendor	Cat. no.	Volume (antibody + flow wash)
IgG kappa (isotype control)	BD Biosciences	557783	2.5 µL + 7.5 µL
IFNγ	BioLegend	502516	2.5 µL + 7.5 µL
IL-4	BioLegend	500818	2.5 µL + 7.5 µL
IL-8	RD systems	IC208R-100UG	2.5 µL + 7.5 µL
IL-10	BioLegend	501412	2.5 µL + 7.5 µL
NF-κB p65 (pS529)	BD Biosciences	558422	0.5 µL + 9.5 µL
p44/42 MAPK (pT180/182)	Cell signaling	4375	0.5 µL + 9.5 µL
p90RSK (pS380)	Cell signaling	13575	1 µL + 9 µL
S6-ribosomal protein (pS235/236)	Cell signaling	4851	0.5 µl + 9.5 µL
TNFα	BioLegend	502916	2.5 µL + 7.5 µL
12. Prepare antibodies for intracellular- and cytokine-staining in a 96-well V-bottom plate by pipetting one antibody/flow wash mix per well (see Table 4)
13. Add 40 µL of cell suspension to each well. Mix well by pipetting up and down 2–3 times
14. Leave the plate for 30 min at RT in the dark
15. Wash the cells twice with flow wash
16. Centrifuge at 500 x *g* for 5 min at RT. Discard the supernatant
17. Resuspend the cells in 150 µL flow wash and leave the plate at 4 °C in the dark until analysis

### Intracellular antigen staining of anti-IgM stimulated samples (Arm b)

1. Transfer the samples from - 80 °C to a box of ice
2. Wash the cells three times with flow wash
  **NOTE**: It is important to add flow wash in excess to see the cell pellet, e.g., add 1.5 mL flow wash to the barcoded cell population.
3. Centrifuge at 500 x *g* for 5 min at RT. Discard the supernatant
  **NOTE:** Collect the Perm Buffer III which contains ethanol for appropriate waste disposal.
4. Prepare the antibody mix for anti-CD16 staining according to Table 3 in a 1.5 mL tube. Add 10 µl Brilliant Stain Buffer and adjust the total volume to 50 µL with flow wash. Add the surface marker mix to the cell pellet and mix by pipetting up and down 2–3 times
5. Leave for 30 min at RT in the dark
6. Wash the cells twice with flow wash
7. Resuspend the cells in 45 μl flow wash. Add 5 μl anti-FoxP3 to the suspension and mix by pipetting up and down 2-3 times
8. Leave for 30 min at RT in the dark
9. Wash the cells twice with flow wash
10. Centrifuge the cells at 500 x *g* for 5 min at RT. Discard the supernatant
11. Resuspend the cells in 40 µl flow wash per intracellular antibody to be studied (i.e., 1280 µl if using the panel of 32 antibodies listed in Table 5)

**Table 5:** Antibody panel for (phospho)protein profiling

Antibody (AF647-conjugated)	Vendor	Cat. no.	Volume (antibody + flow wash)
IgG Kappa (isotype control)	BD Bioscience	557783	5 µL + 5 µl
AKT (pS473)	Cell Signaling	4075	2 µL + 8 µL
AKT(pT308)	Cell signaling	3375	0.5 µL + 9.5 µL
Bcl-2	Cell signaling	82655	1 µl + 9 µL
Bcl-2 (pS70)	BD Biosciences	562531	1.25 µl + 8.75 µL
Bcl-xL	Cell signaling	86387	1 µL + 9 µL
Bim	Cell signaling	10408	1 µL + 9 µL
Btk (pY223)/Itk (pY180)	BD Biosciences	564846	1 µL + 9 µL
Btk (pY551) & p-Itk (pY511)	BD Biosciences	558134	5 µL + 5 µL
Mcl-1	Cell signaling	78471	1 µL + 9 µL
MEK1 (pS218)/MEK2 (pS222)	BD Biosciences	562460	1.25 µL + 8.75 µL
MEK1 (pS298)	BD Biosciences	560043	5 µL + 5 µL
mTOR (pS2448)	BD Biosciences	564242	1.25 µL + 8.75 µL
NF-κB p65 (pS529)	BD Biosciences	558422	0.5 µL + 9.5 µL
p38 MAPK (pT180/182)	Cell signaling	4552	1 µL + 9 µL
p44/42 MAPK (pT202/Y204)	Cell signaling	4375	0.5 µL + 9.5 µL
p53 (pS37)	BD Biosciences	560280	5 µL + 5 µL
p90RSK (pS380)	Cell signaling	13575	1 µL + 9 µL
PLC-γ2 (pY759)	BD Biosciences	558498	5 µL + 5 µL
Rb (pS807/S811)	BD Biosciences	558590	2 µL + 8 µL
S6-ribosomal protein (pS235/236)	Cell signaling	4851	0.5 µL + 9.5 µL
SAPK/JNK (pT183/Y185)	Cell signaling	9257	1.25 µL + 8.75 µL
STAT1 (pS727)	BD Biosciences	560190	5 µL + 5 µL
STAT1 (pY701)	BD Biosciences	612597	5 µL + 5 µL
STAT3 (pS727)	BD Biosciences	558099	2.5 µL + 7.5 µL
STAT3 (pY705)	BD Biosciences	557815	4 µL + 6 µL
STAT5 (pY694)	BD Biosciences	612599	5 µL + 5 µL
STAT6 (pY641)	BD Biosciences	562079	5 µL + 5 µL
SYK (pY525/526)	Cell signaling	12081	1 µL + 9 µL
TBK1 (pS172)	BD Biosciences	558603	1.25 µL + 8.75 µL
Tyrosine (pY100)	Cell signaling	9415	1 µL + 9 µL
ZAP70/SYK (pY319/Y352)	BD Biosciences	557817	5 µL + 5 µL
12. Prepare antibodies for intracellular staining in a 96-well V-bottom plate by pipetting one antibody/flow wash mix per well (see Table 5)
13. Add 40 µL of the cell suspension to each well. Mix well by pipetting up and down 2–3 times
14. Leave for 30 min at RT in the dark
15. Wash the cells twice with flow wash
16. Centrifuge the cells at 500 x *g* for 5 min at RT. Discard the supernatant
17. Resuspend the cells in 150 µL flow wash and leave the plate at 4 °C in the dark until analysis

### Preparation of compensation controls

Prepare compensation controls for the antibody-conjugated fluorochromes in parallel with the antibody staining. Use compensation beads according to the vendor’s instructions. Compensation controls for the live/dead fixable dye are prepared using a mix of live and dead cells.

### Flow cytometry analysis

**NOTE:** The experiment can be run on a flow cytometer with a plate loader. The flow cytometer must be equipped with lasers/filters that can detect the fluorochromes included in the antibody panels, i.e., a BD FACSymphony A5 cytometer (BD Biosciences) or a Cytek 5 L Aurora (Cytek Biosciences, Fremont, California, USA) with 355 nm, 405 nm, 488 nm, 561 nm and 640 nm lasers.

1. Optimize the FSC and SSC voltages with the unstained control (“Thawing and preparation of PBMCs”, d). Use a fully stained sample to inspect whether a signal in any channel is going off-scale. Use Cytek Assay Settings whenever possible, if using spectral flow. Optimized photomultiplier tube (PMT) voltages for fluorescence labels on conventional flow cytometers can be obtained by voltration^21^
2. Run single-color controls and calculate either the compensation (conventional flow) or the spectral unmixing matrix (spectral flow). For spectral unmixing, test whether so-called *Autofluorescence extraction* improves resolution
3. Run the samples. The flow rate should be low enough to not significantly decrease resolution

### Gating strategy and data analysis

**NOTE:** Multiple approaches can be taken to analyzing high-parameter single-cell data, as reviewed elsewhere^22,23^. Below, we describe an approach to manual gating which allows for separation of barcoded cell populations, identification of cellular subsets, and evaluation of (phospho)protein profiles.

1. Import the Flow Cytometry Standard (FCS) data files from the experiment to a flow cytometry analysis software, i.e., FlowJo (Ashland, Oregon, USA) or Cytobank (Mountain View, California, USA)
2. Gating strategy:

**Identification of lymphocytes and FCB populations**

1. Select events collected under consistent flow rate signal by plotting time *versus* a fluorochrome on the UV laser (e.g. anti-CD3) (Fig. 2a)

**Fig. 2:**
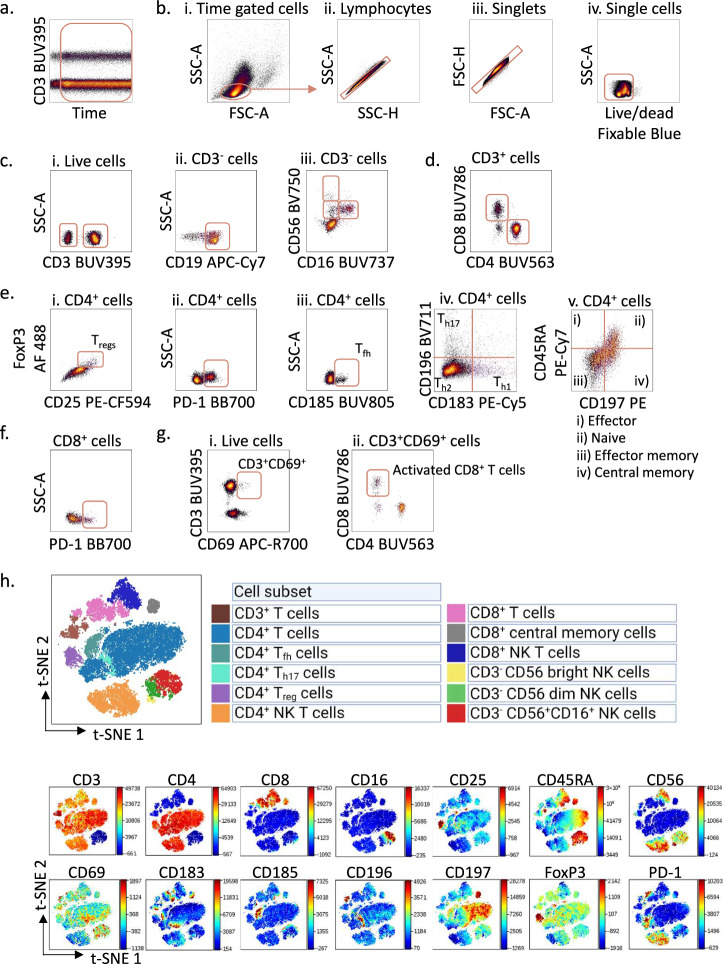
Gating strategy for immunophenotyping of peripheral blood mononuclear cells from healthy donors. **a** Peripheral blood mononuclear cells (PBMCs) from a healthy donor were stained with surface markers and analysed according to the protocol using a Cytek 5 L Aurora instrument. To control for a stable flow stream, the cells were analysed using the time parameter. Here, the cells were gated on signal from the UV laser (anti-CD3) *versus* time. **b**. The time-gated cells from **a**. were selected and lymphocytes were gated in an SSC-A *versus* FSC-A density plot (i), then on lymphocyte singlets in SSC-A *versus* SSC-H (ii) and FSC-H *versus* FSC-A (iii) plots, before live cells were identified in an SSC-A *versus* Live/dead density plot (iv). **c** Live cells (i) gated in **b**. were selected as active population and gated in an SSC-A *versus* CD3 density plot; then on CD3^-^ population, CD19^+^ B cells were gated in an SSC-A versus CD19 density plot (ii) and natural killer (NK) cells were gated in a CD56 *versus* CD16 density plot (iii). **d** CD4^+^ and CD8^+^ cells were gated from the CD3^+^ cells identified in **c**. **e** In the CD4^+^ population, regulatory T-cells (T_regs_) were identified in a FoxP3 *versus* CD25 density plot (i); PD1^+^CD4^+^ T cells were gated in an SSC-A *versus* PD-1 density plot (ii); T follicular helper cells (T_fh_) were gated in an SSC-A *versus* CD185 density plot (iii); T_h1_, T_h2_, T_h17_ were gated in a CD196 *versus* CD183 density plot (iv); naïve T cells, effector T cells, effector memory T cells, and central memory T cells were gated in a CD45RA *versus* CD197 density plot (v). **f** CD8^+^ T cells gated in **d**. were selected and PD-1^+^CD8^+^ T cells were gated in an SSC-A *versus* PD-1 density plot. **g** Live cells gated in **b**. were selected and CD3^+^CD69^+^ cells were gated in a CD3 *versus* CD69 density plot (i), activated CD8^+^ T cells were then identified in a CD8 *versus* CD4 density plot (ii). **h** Non-B cell subsets were visualized in a two-dimensional t-SNE (t-distributed stochastic Neighbour Embedding) plot (top) generated from CD19^-^ cells and gated based on the expression of different markers (bottom). The data were analysed in Cytobank.
2. Select lymphocytes by plotting SSC-A *versus* FSC-A in a density dot plot (Fig. 2b, i)
3. Display the lymphocytes. Plot SSC-A *versus* SSC-H and gate the singlets (Fig. 2b, ii). Double gate on singlets by plotting FSC-H *versus* FSC-A (Fig. 2b, iii)
4. Display singlets. Plot SSC-A *versus* Live/Dead Fixable Blue and gate live cells (Fig. 2b, iv)
5. Display live cells and gate for barcoded cells (see Fig. 1b):
  - Plot Pacific orange *versus* Pacific blue in a density plot and select the different FCB populations based on their staining intensity

**Immunophenotyping: Gating of CD3- populations**

1. Clone the experiment (including compensation, file panels and FCB populations from unstimulated samples)
2. Plot SSC-A *versus* CD3 in a density plot and gate on CD3^+^ and CD3^-^ cells. Tailor per file to assure correct gating for each FCB population (Fig. 2c, i)
3. Display CD3^-^ cells (Fig. 2c, i). Plot SSC-A *versus* CD19 in a density plot and gate for CD3^-^CD19^+^ B cells (Fig. 2c, ii)
4. Display CD3^-^ cells (Fig. 2c, i). Plot CD56 *versus* CD16 in a density plot and gate for natural killer (NK) cells (Fig. 2c, iii):
  - CD16^-^CD56^bright^ NK cells
  - CD16^-^CD56^dim^ NK cells
  - CD16^+^CD56^+^ NK cells

**Immunophenotyping: Gating of T-cell subsets**

1. Display CD3^+^ cells (Fig. 2c, i). Plot CD8 *versus* CD4 in a density plot and gate on CD4^+^CD8^-^ and CD4^-^CD8^+^ T cells (Fig. 2d**)**
2. Display CD4^+^ cells. Plot FoxP3 *versus* CD25 in a density plot and gate on CD25^+^FoxP3^+^ T regulatory (T_reg_) cells (Fig. 2e, i)
3. Display CD4^+^ cells. Plot SSC-A *versus* PD-1 in a density plot and gate on PD-1^+^CD4^+^ T cells (Fig. 2e, ii)
4. Display CD4^+^ cells. Plot SSC-A *versus* CD185 (CXCR5) in a density plot and gate on T follicular helper (T_fh_) cells (Fig. 2e, iii)
5. Display CD4^+^ cells. Plot CD196 *versus* CD183 (CXCR3) in a density plot and gate on T_h1_, T_h2_, and T_h17_ cells (Fig. 2e, iv)
6. Display CD4^+^ cells. Plot CD45RA *versus* CD197 (CCR7) in a density plot and gate on (Fig. 2e, v):
  i. CD45RA^+^CD197^-^ effector CD4^+^ T cells
  ii. CD45RA^+^CD197^+^ naïve CD4^+^ T cells
  iii. CD45RA^-^CD197^-^ effector memory CD4^+^ T cells
  iv. CD45RA^-^CD197^+^ central memory CD4^+^ T cells
7. Display CD8^+^ cells. Plot SSC-A *versus* PD-1 in a density plot and gate on PD-1^+^CD8^+^ T cells (Fig. 2f)
8. Display live cells. Plot CD3 *versus* CD69 in a density plot and gate on CD3^+^CD69^+^ cells (Fig. 2g, i)
9. Display CD3^+^CD69^+^ cells. Plot CD8 *versus* CD4 in a density plot and gate on Activated CD8^+^ cells (Fig. 2g, ii)
10. Additional activation, exhaustion, and subset markers can be investigated with a similar strategy

**(Phospho)protein profiling**

1. Display one cell population from the FCB gate and the desired cell type (i.e., B cells)
2. Plot the (phospho)protein-antibody channel (AF647) against the FCB channel to display (phospho)protein events
3. Calculate (phospho)protein-signals as median fluorescent intensity (MFI). Subtract the corresponding IgG kappa isotype control signal to remove background signal. Normalize the signals in the sample to the signals in the internal control:

$${\rm{Z}}({\rm{Si}})=\frac{[{{\rm{S}}}_{({\rm{phospho}}){\rm{protein}}({\rm{i}})}-{{\rm{S}}}_{{\rm{isotype}}{\rm{control}}({\rm{i}})}]}{[{{\rm{S}}}_{({\rm{phospho}}){\rm{protein}}({\rm{c}})}-{{\rm{S}}}_{{\rm{isotype}}{\rm{control}}({\rm{c}})}]}$$

where S is signal, (i) is sample, and (c) is internal control

### Applications of the protocol

**Fig. 3:**
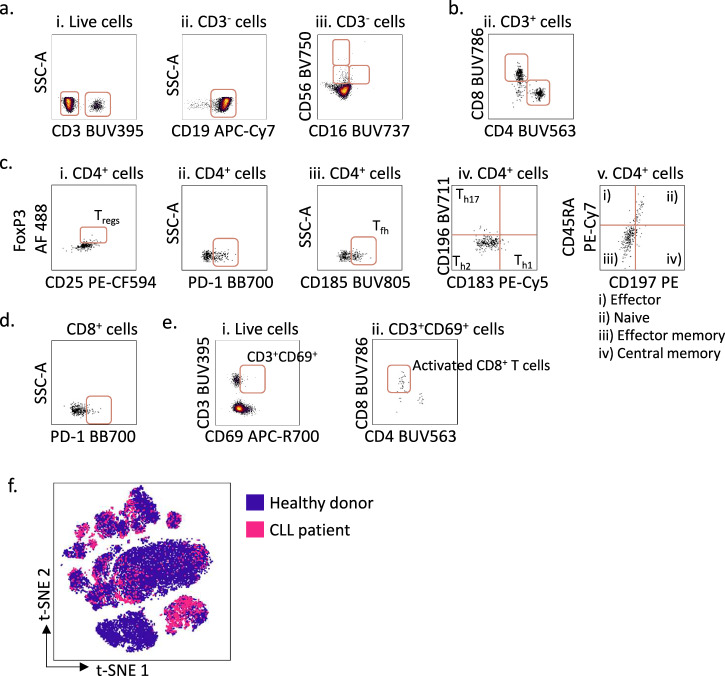
Gating strategy for immunophenotyping of peripheral blood mononuclear cells from CLL patients. **a** Peripheral blood mononuclear cells (PBMCs) from a CLL patient were stained with surface markers and analysed according to the protocol using a Cytek 5 L Aurora instrument. Live cells were gated as described in Fig. 2a, b. and selected as active population (i). The cells were then gated in an SSC-A *versus* CD3 density plot; then on CD3^-^ population, CD19^+^ B cells were gated in an SSC-A versus CD19 density plot (ii) and natural killer (NK) cells were gated in a CD56 *versus* CD16 density plot (iii). **b** CD4^+^ and CD8^+^ cells were gated from the CD3^+^ cells identified in **a**. **c** In the CD4^+^ population, regulatory T-cells (T_regs_) were identified in a FoxP3 *versus* CD25 density plot (i); PD1^+^CD4^+^ T cells were gated in an SSC-A *versus* PD-1 density plot (ii); T follicular helper cells (T_fh_) were gated in an SSC-A *versus* CD185 density plot (iii); T_h1_, T_h2_, T_h17_ were gated in a CD196 *versus* CD183 density plot (iv); naïve T cells, effector T cells, effector memory T cells, and central memory T cells were gated in a CD45RA *versus* CD197 density plot (v). **d** CD8^+^ T cells gated in **b**. were selected and PD-1^+^CD8^+^ T cells were gated in an SSC-A *versus* PD-1 density plot. **e** Live cells were selected and CD3^+^CD69^+^ cells were gated in a CD3 *versus* CD69 density plot (i), activated CD8^+^ T cells were then identified in a CD8 *versus* CD4 density plot (ii). **f** Non-B cell subsets in PBMCs from a healthy donor (blue) and a CLL patient (pink) were visualized in a two-dimensional t-SNE (t-distributed stochastic Neighbour Embedding) plot generated from CD19^-^ cells and overlaid. The data were analysed in Cytobank.

The main steps of the multi-parameter immunophenotyping with single-cell (phospho)protein profiling protocol are illustrated in Fig. 1. Here, we applied the protocol to PBMCs from CLL patients and age-matched healthy donors. The cells were stimulated with anti-IgM for 5 min to activate BCR signaling (Table 1), as indicated in Arm b of Fig. 1a. Two-dimensional barcoding was performed by combining two barcoding dyes at three different dilutions (Table 2 and Fig. 1b), resulting in a barcoding matrix with 9 samples. After labeling, the barcoded samples were combined, stained with antibodies to detect surface and intracellular markers (Tables 3 and 5), and run as one experiment on the flow cytometer. The individual samples can be deconvoluted during the data analysis, and cellular populations and markers are identified by manual gating (Figs. 2 and 3).

**Fig. 4:**
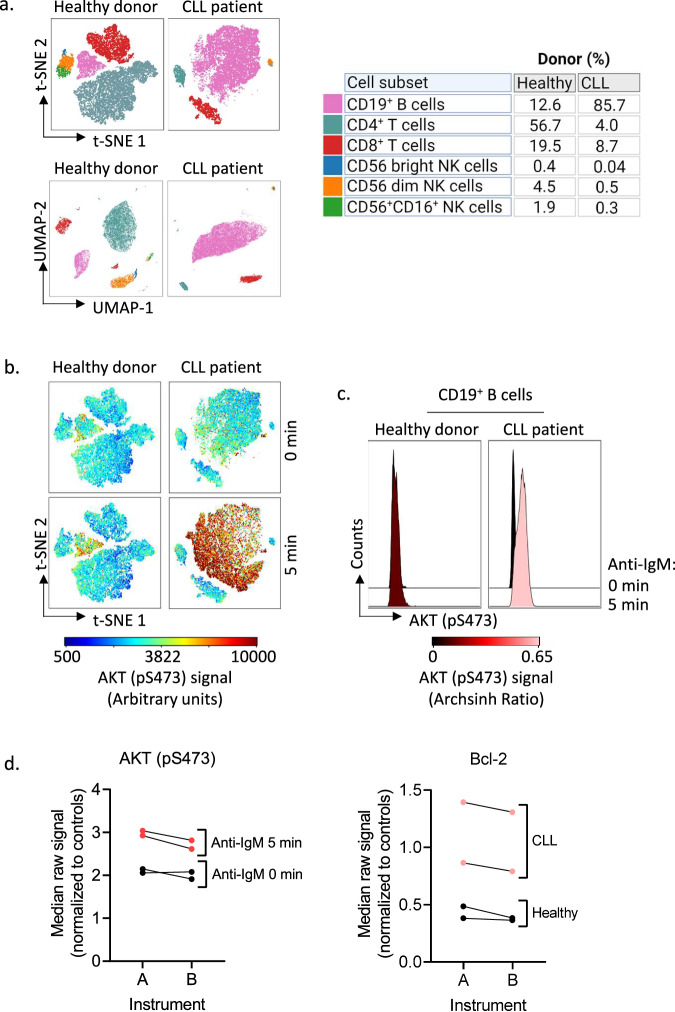
Immunophenotyping and (phospho)protein profiling of peripheral blood mononuclear cells from a healthy donor and a CLL patient. **a** Immunophenotyping data can be visualized in two-dimensional maps. The upper panels show t-SNE (t-distributed stochastic Neighbour Embedding) plots generated from peripheral blood mononuclear cells (PBMCs) samples from a healthy donor and a CLL patient. The plots were generated based on cell surface markers for B cells, T cells and NK cells listed to the right in the figure. The lower panels show UMAPs (Uniform Manifold Approximation and Projection) generated from the same donors based on the same surface markers. Indicated markers were gated based on the signal intensity of each marker in the two-dimensional maps. **b** PBMCs from a healthy donor and a CLL patient were stimulated for 5 min with anti-IgM. t-SNE plots were generated based on the cell surface markers listed in **a**. The signal intensity for AKT (pS473) was used to overlay the t-SNE plots to identify the cell population with signal expression. **c** The experiment was performed as described in **b**. The AKT (pS473) signals in CD19^+^ B cells are shown as archsinh ratio and presented as histograms. **d** The experiment was performed as described in **b** on two different instruments (A; BD FACSymphony A5, and B; Cytek 5 L Aurora). The left panel shows the AKT (pS473) signal in unstimulated and anti-IgM stimulated CD19^+^ B cells from two healthy donors. The right panel shows the Bcl-2 signal in unstimulated CD19^+^ B cells from two CLL (pink) and healthy (black) donor samples. All signals are shown as median raw signal normalized to internal control and IgG kappa isotype. The data were analysed in Cytobank.

To visualize single cells as points and clusters in a two-dimensional plot, we generated t-distributed stochastic neighbor embedding (t-SNE) plots (Figs. 2h, 3f, and 4a) in addition to uniform manifold approximation and projection (UMAP) plots (Fig. 4a). Figure 4a shows the differences in immune cell composition between a healthy donor and a CLL patient, generated based on the surface markers listed in the table to the right. The cell subsets are gated on the surface marker intensity on the clusters in the plots. This approach takes advantage of the computational clustering in the t-SNE or UMAP algorithms rather than relying on manual gating. While the t-SNE plot is commonly used to reveal local data structure, UMAPS preserves more of the global structure and is shown to perform better on large-scale datasets with more information on intercluster relationships^24,25^. Because of this, the distance between the clusters in the UMAP plot is larger than in the t-SNE plot (Fig. 4a).

As expected, the CLL sample was dominated by CD19^+^ B cells, which are the tumor cells (Fig. 4a). Furthermore, we confirmed that the balance between CD4^+^ and CD8^+^ T cell subsets was inverted in PBMCs from the CLL patient, with CD8^+^ T cells being in excess^26^. Since the B-cell population is dominating in CLL PBMCs, it can be challenging to study smaller cell populations such as T_regs_ and naïve/effector subsets in CLL patient samples (Fig. 3a–e). The altered distribution of non-B cells in PBMCs from a CLL patient sample is illustrated in the overlaid t-SNE plot with PBMCs from a healthy donor in Fig. 3f.

In our protocol, immunophenotyping is combined with single-cell (phospho)protein profiling so that cell signaling can be studied in distinct immune subsets. In Fig. 4b, we overlaid the t-SNE plots shown in Fig. 4a with the AKT (pS473) signal detected by the (phospho)protein profiling. The CD19^+^ B-cell cluster in the CLL patient sample showed a clear increase in AKT (pS473) signal in response to anti-IgM stimulation (5 min) compared to both the corresponding unstimulated (0 min) control sample and the stimulated CD19^+^ B cell cluster in the sample from the healthy donor (Fig. 4b). This result was confirmed when the AKT (pS473) signals in CD19^+^ gated B cells were visualized as histograms (Fig. 4c). The other immune cell subsets did not show increased AKT (pS473) signal in response to anti-IgM stimulation, confirming that the stimulation is specific to B cells (Fig. 4b).

**Fig. 5:**
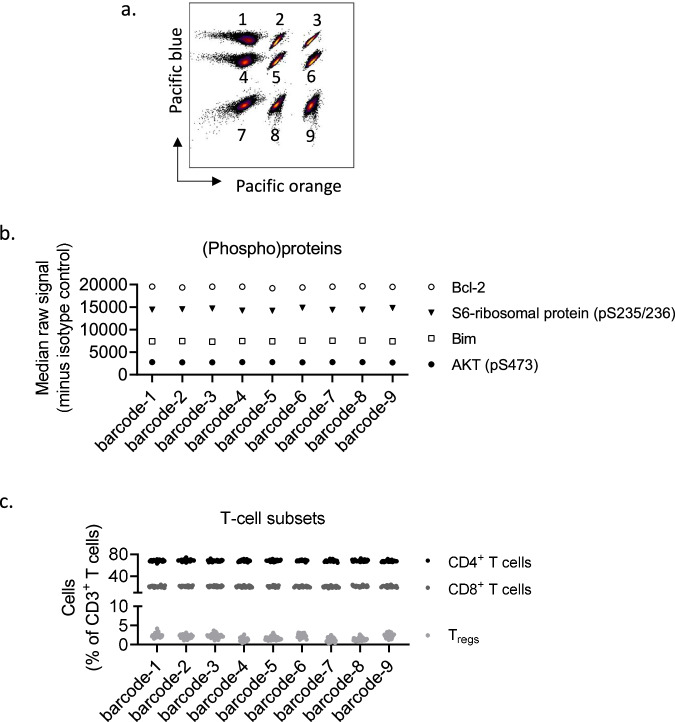
(Phospho)protein profiles and immunophenotypes in barcoded versus non-barcoded cells. **a** Peripheral blood mononuclear cells (PBMCs) from one chronic lymphocytic leukemia (CLL) patient were fixed and barcoded as indicated. **b** The barcoded cells from **a** were combined, permeabilized, and stained with surface markers and antibodies against (phospho)proteins. The experiment was analysed according to protocol using a Cytek 5L Aurora instrument. The graph shows the raw median signal minus the signal of the isotype control for 4 of 31 (phospho)proteins in CD19^+^ B cells of the indicated cell populations. **c** The graph shows the percentage of CD4^+^ T cells, CD8^+^ T cells, and T_regs_ in the CD3^+^ T cell populations from the experiment described above. The points represent phenotype data from 31 replicates obtained from the wells stained with individual (phospho)protein antibodies. The data were analysed in Cytobank.

To study whether the (phospho)protein profiles would be impacted by the type of instrument used for the analysis, we ran the same samples on two different instruments (A; BD FACSymphony A5, and B; Cytek 5L Aurora). As shown in Fig. 4d, we observed very low variability between the instruments. The minor reduction in signal detected by instrument B relative to instrument A is likely due to the automatic removal of autofluorescence by this instrument only (Fig. 4d). In addition, we tested whether (phospho)protein profiles or immunophenotypes were affected by fluorescent cell barcoding. PBMCs from one CLL patient were stained with a barcode matrix (Fig. 5a). We observed that both (phospho)protein signals and immunophenotypes were highly consistent within the barcode matrix (Fig. 5b, c), as expected.

Taken together, these results demonstrate that our combined immunophenotyping and (phospho)protein profiling protocol is high-throughput and robust with high resolution.

## Discussion

The treatment landscape of hematologic malignancies is constantly evolving. This evolution challenges existing prognostic factors, risk scores, and treatment algorithms. In CLL, unmutated IGVH and *TP53* aberrations are historically associated with a poor prognosis^27^. However, with the introduction of targeted therapies, including B-cell lymphoma-2 (Bcl-2) and Bruton’s Tyrosine Kinase (BTK) inhibitors, these factors may show reduced prognostic value^28^. This does not mean that the targeted therapies are curative. Rather, there is a need for novel biomarkers that can stratify responders and non-responders to the new therapies and guide optimal treatment strategies for the individual patient.

Functional biomarkers such as drug sensitivity screens, BH3 profiles, and (phospho)protein profiles have demonstrated pre-clinical and clinical value in predicting drug responses and guiding clinical decision-making^7,15,16,29–34^. The implementation of functional biomarkers in clinical trials underscores the motivation to move functional precision medicine towards routine clinical practice^11,35^. The protocol that we present here may be applied to identify biomarker signatures that can stratify cancer patients.

High-parameter single-cell technologies have shown explosive development in recent years. More advanced instrumentation and an increasing number of available fluorochromes enable the collection of large data-sets in a single experiment. This introduces the need for more advanced data handling and -analysis tools. Manual gating strategies that we have presented here are tailored to the user’s antibody panel and are usually applied for hypothesis-driven analysis. While this gating strategy serves its purpose, the data generated by high-parameter single-cell analyses are generally too complex for manual gating alone. Furthermore, manual gating can be highly time-consuming and may introduce variability and bias due to the subjective nature of the approach^36^. Computational flow cytometry tools have therefore been developed for the analysis, visualization, and interpretation of these types of data^36–38^. Application of automated gating strategies allows for non-hypothesis-driven discoveries in addition to more standardized and reproducible procedures. Available data analysis tools have been subject to comparative evaluations and shown to be accurate and reliable^39,40^.

When multiple experiments are performed over a longer time span, the user is encouraged to include an internal biological control^41,42^. Examples of biological controls are samples with a tested and known protein expression or known loss of antigen expression (internal negative control), or vials from the same batch of PBMCs that are thawed and stained on each assay date. Because the control cells are exposed to identical conditions as the experimental samples, they can be used as a reference or for normalization of the data.

The protocol we have presented here may serve as a guideline for the use and further development of assays to study immunophenotypes and cell signaling at single-cell resolution. The protocol can be used to characterize phenotypes of normal and malignant cells, to understand benign and malignant hematology, and it has the potential to identify biomarkers that may be used to guide clinical decision-making.

## Methods

### Patient material and ethical considerations

Buffy coats from healthy blood donors were received from the Oslo University Hospital Blood Centre, Oslo, Norway. Blood samples from CLL patients were received from the Department of Haematology, Oslo University Hospital, Norway. All donors signed a written informed consent prior to sample collection. The study was approved by the Regional Committee for Medical and Health Research Ethics of South-East Norway (2016/947). The research on human blood was carried out in accordance with the Declaration of Helsinki. Isolation of PBMCs from buffy coats or blood samples was performed as previously described^5,14^. The cells were cryopreserved as previously reported^43^. Unique biological material cannot be distributed.

### Reporting summary

Further information on research design is available in the Nature Research Reporting Summary linked to this article.

### Supplementary information

REPORTING SUMMARY

## Data Availability

The data that support the findings of this study are available from the corresponding author (sigrid.skanland@ous-research.no) upon reasonable request.

## References

[1] Anderson NM, Simon MC (2020). The tumor microenvironment. Curr. Biol..

[2] Skånland SS, Mato AR (2021). Overcoming resistance to targeted therapies in chronic lymphocytic leukemia. Blood Adv..

[3] Aronson JH, Skånland SS, Roeker LE, Thompson MC, Mato AR (2022). Approach to a patient with ‘double refractory’ chronic lymphocytic leukemia: ‘Double, double toil and trouble’ (Shakespeare). Am. J. Hematol..

[4] Svanberg R, Janum S, Patten PEM, Ramsay AG, Niemann CU (2021). Targeting the tumor microenvironment in chronic lymphocytic leukemia. Haematologica.

[5] Myhrvold IK (2018). Single cell profiling of phospho-protein levels in chronic lymphocytic leukemia. Oncotarget.

[6] Skånland SS (2020). An in vitro assay for biomarker discovery and dose prediction applied to ibrutinib plus venetoclax treatment of CLL. Leukemia.

[7] Skånland SS (2022). Functional testing of relapsed chronic lymphocytic leukemia guides precision medicine and maps response and resistance mechanisms. An index case. Haematologica.

[8] Blix ES (2012). Phospho-specific flow cytometry identifies aberrant signaling in indolent B-cell lymphoma. BMC Cancer.

[9] Myklebust JH (2017). Distinct patterns of B-cell receptor signaling in non-Hodgkin lymphomas identified by single-cell profiling. Blood.

[10] Flobak Å, Skånland SS, Hovig E, Taskén K, Russnes HG (2022). Functional precision cancer medicine: drug sensitivity screening enabled by cell culture models. Trends Pharmacol. Sci..

[11] Letai A, Bhola P, Welm AL (2022). Functional precision oncology: Testing tumors with drugs to identify vulnerabilities and novel combinations. Cancer Cell.

[12] Williams ST (2022). Precision oncology using ex vivo technology: a step towards individualised cancer care?. Expert Rev. Mol. Med..

[13] Skånland, S. S., Karlsen, L. & Taskén, K. B cell signaling pathways - new targets for precision medicine in CLL. *Scand. J. Immunol*. e12931 (2020) 10.1111/sji.12931. 10.1111/sji.12931 32640099

[14] Skånland SS (2018). Phospho flow cytometry with fluorescent cell barcoding for single cell signaling analysis and biomarker discovery. J. Vis. Exp..

[15] Yin Y (2022). Functional testing to characterize and stratify PI3K inhibitor responses in chronic lymphocytic leukemia. Clin. Cancer Res..

[16] Melvold K (2022). Mcl-1 and Bcl-xL levels predict responsiveness to dual MEK/Bcl-2 inhibition in B-cell malignancies. Mol. Oncol..

[17] Giliberto M (2022). Mutational analysis and protein profiling predict drug sensitivity in multiple myeloma cell lines. Front. Oncol..

[18] Krutzik PO, Clutter MR, Trejo A, Nolan GP (2011). Fluorescent cell barcoding for multiplex flow cytometry. Curr. Protoc. Cytom..

[19] Nooti S, Naylor M, Long T, Groll B, Manu (2023). LucFlow: A method to measure Luciferase reporter expression in single cells. PLoS One.

[20] Behbehani GK (2014). Transient partial permeabilization with saponin enables cellular barcoding prior to surface marker staining. Cytom. Part A J. Int. Soc. Anal. Cytol..

[21] BDBiosciences. *BD FACSDivaTM software Determining Initial PMT Voltages* https://www.bdbiosciences.com/content/dam/bdb/marketing-documents/BD-FACSDiva-Initial-PMT-Voltages.pdf

[22] Chattopadhyay PK, Winters AF, Lomas WE, Laino AS, Woods DM (2019). High-parameter single-cell analysis. Annu. Rev. Anal. Chem..

[23] Sun J, Kroeger JL, Markowitz J (2021). Introduction to multiparametric flow cytometry and analysis of high-dimensional data. Methods Mol. Biol..

[24] Toghi Eshghi S (2019). Quantitative comparison of conventional and t-SNE-guided gating analyses. Front. Immunol..

[25] Becht, E. et al. Dimensionality reduction for visualizing single-cell data using UMAP. *Nat. Biotechnol*. 10.1038/nbt.4314 (2018). 10.1038/nbt.4314 30531897

[26] Man S, Henley P (2019). Chronic lymphocytic leukaemia: the role of T cells in a B cell disease. Br. J. Haematol..

[27] International CLL-IPI working group. An international prognostic index for patients with chronic lymphocytic leukaemia (CLL-IPI): a meta-analysis of individual patient data. *Lancet. Oncol*. **17**, 779–790 (2016). 10.1016/S1470-2045(16)30029-8 27185642

[28] Hallek M, Al-Sawaf O (2021). Chronic lymphocytic leukemia: 2022 update on diagnostic and therapeutic procedures. Am. J. Hematol..

[29] Kornauth C (2022). Functional precision medicine provides clinical benefit in advanced aggressive hematologic cancers and identifies exceptional responders. Cancer Discov..

[30] Malani D (2022). Implementing a functional precision medicine tumor board for acute myeloid leukemia. Cancer Discov..

[31] Montero J (2017). Blastic plasmacytoid dendritic cell neoplasm is dependent on BCL2 and sensitive to Venetoclax. Cancer Discov..

[32] Kuusanmäki H (2023). Ex vivo venetoclax sensitivity testing predicts treatment response in acute myeloid leukemia. Haematologica.

[33] Davids MS (2012). Decreased mitochondrial apoptotic priming underlies stroma-mediated treatment resistance in chronic lymphocytic leukemia. Blood.

[34] Andersen AN (2023). Clinical forecasting of acute myeloid leukemia using ex vivo drug-sensitivity profiling. Cell Rep. methods.

[35] Ayuda-Durán P (2023). Standardized assays to monitor drug sensitivity in hematologic cancers. Cell Death Discov..

[36] Saeys Y, Van Gassen S, Lambrecht BN (2016). Computational flow cytometry: helping to make sense of high-dimensional immunology data. Nat. Rev. Immunol..

[37] Quintelier K (2021). Analyzing high-dimensional cytometry data using FlowSOM. Nat. Protoc..

[38] Todorov H, Saeys Y (2019). Computational approaches for high-throughput single-cell data analysis. FEBS J..

[39] Weber LM, Robinson MD (2016). Comparison of clustering methods for high-dimensional single-cell flow and mass cytometry data. Cytom. Part A J. Int. Soc. Anal. Cytol..

[40] Aghaeepour N (2013). Critical assessment of automated flow cytometry data analysis techniques. Nat. Methods.

[41] Hulspas R, O’Gorman MRG, Wood BL, Gratama JW, Sutherland DR (2009). Considerations for the control of background fluorescence in clinical flow cytometry. Cytom. B. Clin. Cytom..

[42] Mizrahi O, Ish Shalom E, Baniyash M, Klieger Y (2018). Quantitative flow cytometry: concerns and recommendations in clinic and research. Cytom. B. Clin. Cytom..

[43] Hermansen JU, Tjønnfjord GE, Munthe LA, Taskén K, Skånland SS (2018). Cryopreservation of primary B cells minimally influences their signaling responses. Sci. Rep..

